# Toward the favorable consequences of academic motivation and L2 enjoyment for students' willingness to communicate in the second language (L2WTC)

**DOI:** 10.3389/fpsyg.2022.997566

**Published:** 2022-08-22

**Authors:** Guihua Cao

**Affiliations:** School of Foreign Languages, Hubei Engineering University, Xiaogan, China

**Keywords:** willingness to communicate in the second language (L2WTC), academic motivation, L2 enjoyment, L2 acquisition, theoretical evidence

## Abstract

Students' willingness to communicate in the second language (L2WTC) is perceived to be the ultimate goal of L2 acquisition in that high levels of L2WTC facilitate L2 use. For this reason, factors leading learners to higher levels of L2WTC have been widely scrutinized. Yet, the role of psycho-emotional factors like academic motivation and L2 enjoyment in promoting students' L2WTC has remained elusive. Moreover, as existing literature reveals, no inquiry has conceptually reviewed the impacts of these factors on students' L2WTC. To respond to this gap, this conceptual review strived to elucidate the consequences of academic motivation and L2 enjoyment for students' L2WTC. The favorable effects of academic motivation and L2 enjoyment on students' L2WTC levels were shown in the light of empirical and theoretical evidence. Finally, the potential implications of the findings are highlighted.

## Introduction

Students' L2 achievement is tied to their willingness to communicate in the second language (L2WTC). Simply said, those who are reluctant to speak in the target language will fail to achieve desirable language outcomes (Menezes and Juan-Garau, 2015; Joe et al., 2017; Lee et al., 2022). This made students' L2WTC an important concern for teachers in any language learning environment. L2WTC, as Clément et al. (2003) mentioned, pertains to “students' preparedness to enter into discourse at a particular time with a specific person or persons, using an L2” (p. 192). Put simply, L2WTC reflects an individual student's mental readiness to initiate the conversation with others in the second language (Peng, 2015). According to Elahi Shirvan et al. (2019), L2WTC as a “personality-based predisposition” serves an outstanding role in the process of second language acquisition (SLA). They noted that students who are willing to communicate in the target language will effectively proceed with the SLA process. Likewise, Chen et al. (2022) mentioned that L2WTC can dramatically affect the SLA process in that it helps students cultivate their communicative competence.

As positive psychology (PP) has blossomed in the language education domain (Dewaele et al., 2019a; Wang Y. et al., 2021), L2 practitioners' focus has shifted from examining negative psychological factors like unwillingness to communicate (UWTC) to investigating positive psychological factors, notably L2WTC. The centrality of L2WTC in the SLA process has made it appealing for L2 researchers to study the determinants of this construct. That is, a great deal of attention has been devoted to scrutinizing the personal, emotional, and situational determinants of students' willingness to communicate in an L2 context (e.g., Alemi et al., 2013; Elahi Shirvan and Taherian, 2016; Yue, 2016; Liu, 2017; Dewaele and Dewaele, 2018; Reid and Trofimovich, 2018; Zhang et al., 2018; Dewaele, 2019; Lee and Drajati, 2019; Lee and Lee, 2020; Lan et al., 2021; Lee et al., 2021b; Pishghadam et al., 2021b; Wang H. et al., 2021; Song et al., 2022, to cite a few). Yet, the role of L2 enjoyment and academic motivation in predicting students' L2WTC has been narrowly explored (Fallah, 2014; Dewaele, 2019; Lin, 2019; Lee, 2020; Alrabai, 2022). Accordingly, whether academic motivation and L2 enjoyment can determine students' L2WTC is still an open question. To respond to this question, through theoretical and empirical evidence, this conceptual review seeks to describe the consequences of academic motivation and L2 enjoyment for students' L2WTC.

As a potential determinant of L2WTC, student academic motivation refers to “individual students' primary impetus for initiating learning as well as the reason for continuing the prolonged and tedious process of learning” (Ushioda, 2008, p. 21). In the SLA domain, academic motivation pertains to the L2 learners' internal motive to commence and continue the lengthy process of second language acquisition (Hiromori, 2009). Academic motivation, according to Syed and Kuzborska (2020), urges L2 learners to willingly communicate with others through the second language. In fact, academic motivation positively affects an individual student's decision to speak in the second language. Academic motivation, as Wen (2022) mentioned, also prompts L2 learners to do their utmost to acquire the second language. Likewise, Wang (2022) declared that academic motivation pushes L2 learners to invest a huge amount of time, energy, and attempt in mastering the target language.

Another potential determinant of students' WTC in an L2 context is L2 enjoyment, which pertains to the extent to which the L2 learning experience brings about joy, pleasure, and happiness in classroom settings (Goetz et al., 2006). For Dewaele and MacIntyre (2016), L2 enjoyment is “a complex emotion, capturing interacting dimensions of the challenge and perceived ability that reflects the students' drive for success in the face of difficult tasks” (p. 216). As pointed out by Dewaele et al. (2018), L2 enjoyment emerges as students' academic needs and expectations are fully satisfied throughout the learning process. According to Lee (2020), learners who experience enjoyment inside the L2 classes aspire to constantly participate in classroom interactions. This helps them become more proficient and fluent in using the target language (Khajavy et al., 2016; Darasawang and Reinders, 2021). Furthermore, as Guo (2021) pinpointed, L2 enjoyment prompts learners to joyfully participate in the SLA process. This, in turn, enables them to attain the expected language achievement (Jin and Zhang, 2018).

Considering the pivotal function of L2 enjoyment and academic motivation in the language acquisition process, several L2 scholars have scrutinized the academic consequences of these variables (e.g., Li et al., 2016; Li, 2020, 2021; Zhang et al., 2020; Fathi and Mohammaddokht, 2021; Zoghi, 2021, among others; Dewaele and Li, 2021). Notwithstanding, scant research attention has been given to the pedagogical consequences of these variables for students' L2WTC (Fallah, 2014; Dewaele, 2019; Lin, 2019; Lee, 2020; Alrabai, 2022). Additionally, no study has conceptually reviewed the role of academic motivation and L2 enjoyment in students' L2WTC. To answer these gaps, the current research strives to elucidate the function of these emotional variables in students' L2WTC using empirical and theoretical underpinnings.

## Literature review

### Academic motivation

As a driving force, motivation offers individuals a reason to initiate a given action and persist in it (Crookes and Schmidt, 1991; Christophel and Gorham, 1995). In language classes, motivation gives students a reason to take up language acquisition and persevere in its long-lasting process (Rotgans and Schmidt, 2012). In this respect, language learners' academic motivation has to do with their motive for undertaking the long, demanding process of language learning (Ushioda and Dörnyei, 2009). As Ryan and Deci (2020) mentioned, the underlying motive of an individual learner to take part in the language learning process may be either internal or external. Accordingly, learners' academic motivation includes two distinct dimensions: “*inner motivation*” and “*outer motivation*” (Ryan and Deci, 2020). Inner motivation, according to Ryan and Deci (2020), pertains to “activities done for their own sake or for their inherent interest and enjoyment” (p. 2). Extending this to the SLA domain, learners' inner motivation is concerned with their decision to acquire a second language for its own sake (Papi and Hiver, 2020). Outer motivation, as Ryan and Deci (2020) noted, refers to “behaviors done for reasons other than their inherent satisfactions” (p. 3). In this regard, learners' outer motivation deals with their intention to take part in the language acquisition process for the sake of extrinsic rewards (Al-Hoorie et al., 2022). Taken together, inner and outer academic motivation are what prompt students to engage in the learning experience (Ryan and Deci, 2017).

Language learners' inner and outer motivation are believed to remarkably predict their classroom engagement, which, in turn, results in desirable language scores (Froiland and Worrell, 2016; Wu, 2019; Pishghadam et al., 2021b). Because of this, the internal and external sources of learners' academic motivation have always been at the forefront of researchers' attention. That is, numerous researchers have delved into the internal and external determinants of learners' academic motivation (e.g., Mojavezi and Tamiz, 2012; Pan, 2014; Cheon and Reeve, 2015; Estepp and Roberts, 2015; Kiefer et al., 2015; Song et al., 2015; Furlich, 2016; Oqvist and Malmstrom, 2018; Liu, 2021; Pishghadam et al., 2021a, to cite a few). As for the internal determinants, Oqvist and Malmstrom (2018), for instance, assessed the effect of students' self-efficacy on their learning motivation. To do so, 993 school students' viewpoints were surveyed using two pre-developed questionnaires. Considering respondents' viewpoints, researchers discovered that self-efficacy beliefs can significantly affect students' learning motivation. Besides, concerning the external sources of student motivation, Furlich (2016) evaluated the impact of teachers' immediate behaviors on students' academic motivation. To this end, three valid questionnaires, including “Verbal Immediacy Scale”, “Nonverbal Immediacy Scale”, and “Motivation to Learn Questionnaire”, were given to 77 college students. The results of data analysis disclosed the positive influences of teachers' immediacy on students' academic motivation. Later, Pishghadam et al. (2021a) studied the function of teacher stroke in students' motivation. To this aim, 437 university students were recruited from different universities in Iran. Then, to obtain the required data, two valid surveys were distributed among respondents. The examination of respondents' viewpoints revealed that teachers' stroking behaviors positively contribute to students' motivation.

### L2 enjoyment

The notion of enjoyment has been literally defined as “a good feeling coming from breaking through homeostatic limits and stretching beyond oneself to accomplish something new or even unexpected, especially in face of some difficult tasks” (Li et al., 2018, p. 185). Extending this into L2 classes, Mierzwa (2019) conceptualized L2 enjoyment as a desired, activating, and action-focused emotion that captures interrelated facets of challenge and perceived ability, representing language learners' motive for success in the face of learning adversities. Deeply rooted in control-value theory, L2 enjoyment is heavily reliant on the value that learners attribute to the learning activities (Piniel and Albert, 2018). As Zhang and Tsung (2021) mentioned, it also depends on the degree to which learners feel in control of the classroom atmosphere and their language attainments, and the extent to which they attribute their language attainments to their personal endeavors or abilities.

As a prime instance of achievement emotions, L2 enjoyment can prompt learners to reach higher levels of language achievement (Shao et al., 2019). For this reason, many research studies have investigated the predictors of students' enjoyment in an L2 context (e.g., Boudreau et al., 2018; Dewaele and MacIntyre, 2019; Dewaele et al., 2019b; Rezazadeh and Zarrinabadi, 2020; Li et al., 2021; Li, 2022, to cite a few). Dewaele et al. (2019a), for instance, analyzed the role of teachers' traits in predicting Spanish students' enjoyment. To do this, 210 Spanish learners were asked to fill out two pre-designed questionnaires. As a result, a strong, favorable association was found between teachers' traits and students' enjoyment. Moreover, students' enjoyment was found to be strongly predicted by the teachers' characteristics. In their study, Rezazadeh and Zarrinabadi (2020) also examined the predictive power of need for closure and need for cognition. In doing so, three valid surveys were given to 232 Iranian EFL students. The path analysis discovered that both need for closure and need for cognition can directly predict Iranian students' enjoyment in EFL classes. Additionally, in a recent inquiry, Li et al. (2021) inspected the function of emotional intelligence and classroom atmosphere in predicting Chinese students' enjoyment. To accomplish this, three reliable scales were distributed among a large sample of 3,013 Chinese students. As data analysis revealed, Chinese students' enjoyment was positively predicted by emotional intelligence and classroom atmosphere.

### Willingness to communicate in a second language (L2WTC)

The concept of “*Willingness to Communicate (WTC)*” in a general sense refers to an individual's tendency to commence oral communication with others (McCroskey and Baer, 1985; MacIntyre, 1994; MacIntyre and Doucette, 2010). In this sense, one's willingness to communicate in a second language, which is called L2WTC, pertains to his/her inclination to communicate at a given time with others, using a second language (MacIntyre et al., 2003; Pawlak and Mystkowska-Wiertelak, 2015). For MacIntyre (2020), learners' L2WTC is a dynamic psychological variable that may alter as time goes by. He believes that learners' decisions to communicate in an L2 may fluctuate throughout the learning process as a result of personal, interpersonal (Xie and Derakhshan, 2021), and situational variables.

As high levels of L2WTC facilitate L2 use (Yu et al., 2011; Ghonsooly et al., 2012; Lee et al., 2021a), factors that may encourage learners to communicate in the second language have been of great importance to L2 researchers. Because of this, a spate of inquiries have been previously conducted on the predictors of learners' WTC in an L2 context (e.g., Liu, 2017; Dewaele and Dewaele, 2018; Reid and Trofimovich, 2018; Sheybani, 2019; Freiermuth and Ito, 2020; Wang Y. et al., 2021; Ito, 2022, to cite a few). Freiermuth and Ito (2020), for example, studied the role of students' personality and classroom experience in their WTC. For this purpose, a sample of 69 university students was recruited. Then, three questionnaires were administered to participants in order to evaluate their personality, WTC, and perceived classroom experience. The results uncovered the power of students' personalities and previous experiences in predicting their WTC. In another investigation, Wang H. et al. (2021) securitized the function of academic emotions, including boredom, anxiety, and pride in L2 learners' WTC. To examine learners' boredom, anxiety, pride, and WTC in L2 classes, four close-ended scales were distributed among 811 college students. Except for pride, which had a positive impact on students' WTC, all the aforementioned emotions negatively predicted students' WTC inside the classrooms. Additionally, Ito (2022) inspected the impact of general trust on L2 learners' WTC. To this end, two pre-designed surveys were administered to assess university students' general trust and L2WTC. The regression analysis outcomes demonstrated that general trust had a remarkable and positive impact on students' L2WTC.

### Previous research on the role of academic motivation and L2 enjoyment in students' L2WTC

As previously noted in this review, despite the important role of academic motivation and L2 enjoyment in encouraging learners to talk, the influences of these variables on students' L2WTC have sporadically been scrutinized. Put simply, only a few L2 scholars have sought to unravel the impact of these psycho-emotional constructs on learners' L2WTC (Fallah, 2014; Dewaele, 2019; Lin, 2019; Lee, 2020; Alrabai, 2022). As an instance, Fallah (2014) set out to measure the influence of motivation on Iranian students' L2WTC. In doing so, 252 EFL learners were invited to answer two reliable scales. The outcomes of SEM analysis revealed that motivation can noticeably influence students' WTC inside L2 classes. By the same token, Lin (2019) probed the impact of academic motivation on Taiwanese EFL learners' L2WTC. To do so, the academic motivation scale and the L2WTC questionnaire were given to 701 EFL learners who were selected at random from several universities in Taiwan. Scrutinizing participants' answers to the above-mentioned scales, the researcher found that EFL learners' L2WTC can be favorably affected by their academic motivation. Furthermore, Dewaele (2019) investigated the role of academic emotions, including L2 enjoyment, in improving students' L2WTC. Employing two close-ended questionnaires, the researcher examined Spanish EFL learners' academic motivation and their propensity to communicate in the second language. Performing multiple regression analyses, he observed found learners' academic motivation can dramatically increase their L2WTC. Additionally, in a more recent inquiry, Alrabai (2022) analyzed the consequences of L2 enjoyment and learning motivation for students' L2WTC. For this purpose, 328 EFL students were invited to take part in this investigation. Participants' motivation, enjoyment, and L2WTC were inspected using three self-report questionnaires. The results of a partial least SEM analysis showed that both motivation and L2 enjoyment were positive predictors of students' L2WTC. Despite such scholarly attempts, the consequences of academic motivation and L2 enjoyment for students L2WTC are not widely identified, which warrants more empirical and review studies on this subject.

## The role of academic motivation and L2 enjoyment in students' L2WTC: Theoretical basis

**Figure 1:**
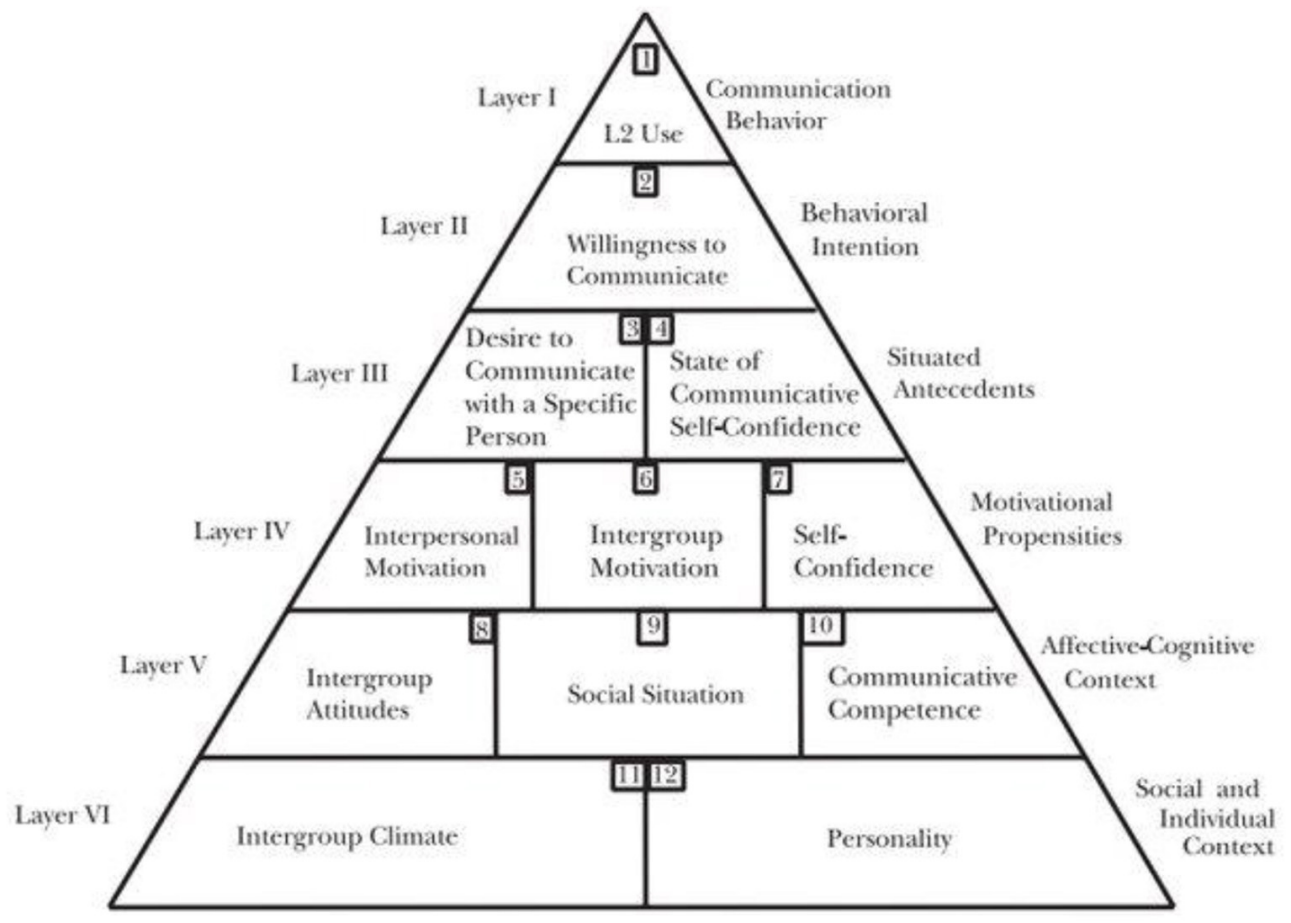
Pyramid model of L2WTC (MacIntyre et al., 1998, p. 546).

The role of learners' academic motivation and L2 enjoyment in their L2WTC level can be clearly illustrated through MacIntyreMacIntyre's et al. (1998) pyramid model of L2WTC (Figure 1).

In their heuristic model, MacIntyre et al. (1998) classified different personal, emotional, and contextual sources of learners' L2WTC into six main layers. These six layers, as Figure 1 demonstrates, are grouped into three lower layers that represent “distal, enduring, and widely applicable influences on L2 communication” and three top layers that indicate “immediate, transient, situation-specific effects on L2WTC at a given moment” (MacIntyre and Wang, 2021, p. 4). With regard to the lowest layer of this conceptual model (Layer 6), an individual learner's intention to communicate in the second language is subject to the intergroup climate (MacIntyre, 1994). That is, an appropriate intergroup climate can urge learners to communicate with their classmates and teachers, using an L2 (MacIntyre et al., 2003). The appropriateness of intergroup climate in a given academic setting highly relies on the extent to which students perceive the learning atmosphere to be joyful, pleasant, and enjoyable (Khajavy et al., 2018). Accordingly, a learning atmosphere conveying a sense of enjoyment can favorably influence L2 learners' decisions to communicate in the target language (MacIntyre and Wang, 2021). Moving up the pyramid, layer 4 reflects the function of motivational propensities in learners' L2WTC level. Putting motivational propensities at the upper layers of the pyramid, MacIntyre et al. (1998) highlighted the role of learners' personal and interpersonal motivation in their L2WTC level. According to them, L2 learners' motivation to learn can enormously influence their propensity to communicate in the target language (MacIntyre, 2007). They believe that in the presence of learning motivation, L2 learners are more inclined to have oral communication in classroom settings. Building upon this conceptual model, both academic motivation and L2 enjoyment appear to be highly influential in L2 learners' decisions to speak.

## Conclusion

The present study reviewed the theoretical and operational definitions of academic motivation, L2 enjoyment, and L2WTC. In this respect, the underlying facets of these constructs were also characterized by referring to their theoretical models. Moreover, prior research on the impacts of academic motivation and L2 enjoyment on students' L2WTC was meticulously reviewed. Additionally, in line with the ultimate goal of this review, the favorable consequences of academic motivation and L2 enjoyment for students' L2WTC were elucidated through empirical and conceptual evidence. Drawing on the existing evidence, one can reasonably conclude that academic motivation and L2 enjoyment can urge L2 learners to communicate in a second language. Put simply, in the presence of these academic emotions, L2 learners are more inclined to communicate inside the classes, using an L2.

This seems to be of great help for all L2 teachers who suffer from their learners' lack of motivation. Given the significance of academic motivation in promoting students' L2WTC, teachers are highly advised to care about their learners, appreciate their efforts, satisfy their educational expectations, and establish intimate relationships with them. These behaviors, in turn, improve learners' academic motivation (Cheon and Reeve, 2015; Estepp and Roberts, 2015). Besides, the review outcomes appear to be helpful to L2 teachers who struggle with their learners' unwillingness to speak in the second language. As L2 enjoyment was found to be influential in students' L2WTC, L2 teachers are required to provide their learners with a calm, stress-free learning atmosphere that offers a sense of pleasure and enjoyment. Furthermore, owing to the significance of L2 enjoyment in students' L2WTC, teacher educators are strongly advised to instruct their teacher students how to provide an enjoyable learning atmosphere for their students.

## Suggestions for future research

As the review of the related literature revealed, there is a paucity of research on the role of academic motivation and L2 enjoyment in students' L2WTC. Simply said, only a few studies have been carried out to unravel the function of these psycho-emotional constructs in students' willingness to communicate inside the L2 classes. Considering this, future empirical inquiries are required to assess the role of academic motivation and L2 enjoyment in students' L2WTC. Furthermore, with respect to the outcomes of this review, the previous studies only employed close-ended questionnaires to elicit participants' viewpoints regarding the role of academic motivation and L2 enjoyment in students' L2WTC. To gather more accurate and comprehensive data, future investigations are strongly advised to use other means of data collection as well.

## Author contributions

The author confirms being the sole contributor of this work and has approved it for publication.

## Funding

This study was sponsored by Major Programs for Philosophical and Social Sciences Research of Higher Learning Institutions of Hubei Province—The Study on the Infiltration of Chinese Traditional Culture in College English Education from the Perspective of the Promotion of Cultural Soft Power (Grant No.: 19ZD056).

## Conflict of interest

The author declares that the research was conducted in the absence of any commercial or financial relationships that could be construed as a potential conflict of interest.

## Publisher's note

All claims expressed in this article are solely those of the authors and do not necessarily represent those of their affiliated organizations, or those of the publisher, the editors and the reviewers. Any product that may be evaluated in this article, or claim that may be made by its manufacturer, is not guaranteed or endorsed by the publisher.
